# Acellular human glans extracellular matrix as a scaffold for tissue engineering: in vitro cell support and biocompatibility

**DOI:** 10.1590/S1677-5538.IBJU.2014.0422

**Published:** 2015

**Authors:** Fernanda M. Egydio, Luiz G. Freitas, Kleber Sayeg, Marcus Laks, Andréia S. Oliveira, Fernando G. Almeida

**Affiliations:** Departamento de Cirurgia, Universidade Federal de São Paulo São Paulo, SP, Brasil; Departamento de Nefrologia Universidade Federal de São Paulo São Paulo, SP, Brasil; Deparamento de Urologia Feminina, Universidade Federal de São Paulo São Paulo, SP, Brasil

**Keywords:** Extracellular Matrix, Materials Testing, Neoplasms

## Abstract

**Objectives:**

Diseases of the genitourinary tract can lead to significant damage. Current reconstructive techniques are limited by tissue availability and compatibility. This study aims to assess if the decellularized human glans can be used as a biomaterial for penile reconstruction.

**Materials and Methods:**

Samples of the glans matrices were descellularized. We evaluate the presence of collagen type I and III, and elastic fibers. Biocompatibility assays were performed to assess the cytotoxic and non-cytotoxic interactions between the acellular matrix and 3T3 cells. The matrices were seeded with mesenchymal stem cells and were assessed for viability and integration of these cells. Biomechanical tests in native tissue, descellularized matrix and seeded matrix were performed to characterize their biomechanical properties.

**Results:**

The tissue architecture of the decellularized matrix of human glans was preserved as well as the maintenance of the biomechanical and biological properties. The analyzes of glans seeded with mesenchymal stem cells revealed the integration of these cells to the matrices, and its viability during two weeks “in vitro”.

**Conclusion:**

The decellularization process did not alter the biological and biomechanical characteristics of the human glans. When these matrices were seeded they were able to maintain the cells integrity and vitality.

## INTRODUCTION

Congenital diseases, cancer, trauma, and other conditions can lead to significant glans damage requiring reconstruction. This is a major challenge because there are great limitations of available compatible grafts due to glans complex anatomical arrangement, and the psychological impact of its absence is usually very important. In addition, complications can occur when tissue reconstruction of glans is performed due to a transient ischemia caused by vascular impairment, which may even evolve to necrosis (1). In fact, all alternative surgical techniques described for tissue reconstruction result in an organ anatomically similar to a penis, but erectile function can almost never be restored, and the results, both functional and aesthetic, are thus considered inadequate (2–5).

This study aims to assess if the decellularized human glans can be used as a biomaterial for penile reconstruction, and whether the decellularization process changes the biomechanical and biological characteristics of the matrices. Furthermore, we analyzed the integration of mesenchymal stem cells into decellularized matrices of human glans.

## MATERIALS AND METHODS

### 

#### Ethical approval

All experimental procedures were approved by the local Research Ethics Committee (0878/09) and conducted in strict conformity with local institutional guidelines and with international standards for the manipulation and care of laboratory animals.

#### Obtaining the glans matrices

The matrices were obtained from the glans penis of human donor tissue for transplantation. Each fragment extended from the coronal region to the urethral meatus, maintaining the dorsal neuro-vascular bundle and was stored in a PBS solution (Phosphate-Buffered Saline) at 4°C, supplemented with penicillin 10,000 IU/L and streptomycin 50 mg/L.

#### Decellularization of human glans matrices

Dissection of the fragments exposed the deep dorsal vein of the penis, which was then isolated for insertion of a catheter through which 1200 mL of distilled water at 4°C was perfused. 500 mL of decellularization solution with 1% Triton X-100 and 0.1% ammonium hydroxide at 4°C was then perfused and the fragments were maintained in this solution for 7 days for complete removal of cellular components. The matrices were then perfused with 600 mL of distilled water and 300 mL of PBS and stored in glycerol 87% at 4°C.

#### Cell Culture of Mesenchymal stem cells

The mesenchymal stem cells were isolated from femurs and tibiae of male Wistar rats ranging in weight from 150-250 g. For the separation of the mononuclear fraction of bone marrow, the material was subjected to the Histopaque protocol (Sigma-Aldrich, St. Louis, MO, USA). After centrifugation at 1800 rpm for 30 minutes, the mononuclear cells were selected from the interface established between the Histopaque and PBS, and were washed with sterile PBS. These cells were then cultured in 25 cm^2^ polystyrene flasks with 5 mL of DMEM low glucose (Dulbecco's Modified Eagle's medium, DMEM Low Glucose - Sigma Chemical Company, St. Louis, USA), supplemented with 10% fetal bovine serum (FBS), and maintained at 37°C, in a humidified 5% (v/v) CO_2_ in air atmosphere for 10 to 15 days. For each cell culture passage, the cells were subjected to trypsin-EDTA (Sigma Chemical Company, St. Louis, USA), seeded, and cultured until to obtain adequate cell number for the experiments.

#### Determination of cellularity

The decellularized glans samples were fixed in 10% buffered formalin, paraffin embedded, sectioned transversely (5μm), and stained with hematoxylin-eosin (H&E). Using a light microscope interfaced with a digital image analysis system, the samples were analyzed to confirm complete removal of the cellular components of the tissue. Transverse cross sections (10μm) of native tissue and decellularized glans matrix were blocked for 10 minutes with a solution containing PBS, 0.2% gelatin, and 0.1% saponin, incubated for 15 minutes with DAPI (4′,6-diamidine-2-phenyl indole dihydrochloride 0.1 mg/mL, Sigma), washed twice with PBS, and finally mounted in buffered glycerol with 0.1 M Tris/HCl, pH 8.8, and 0.01% p-fenilenodiamino. To detect the presence of intact cell nuclei, images were analyzed under a microscope SP5 Confocal Leica TS (200x), NA=0.7.

#### Histology evaluation

To characterize the preservation of biological properties of the decellularized glans matrices, some samples were fixed in 10% buffered formalin, paraffin embedded, sectioned transversely (5μm), and stained using the Verhoff-Von Gieson and Masson's Trichrome methods. Using a light microscope interfaced with a digital image analysis system, these samples were analyzed by assessing the preservation of collagen fibers by Masson Trichrome staining, and elastic fibers by Verhoff-Von Gieson staining, and comparing with the pattern found in the control group that corresponded to the glans tissue.

#### Cytotoxicity and biocompatibility evaluation

Cytotoxicity tests were performed using the direct contact method described by Ciapetti et al. (6) in order to detect potential lethal effects on the cells. A small amount of the decellularized glans samples were put directly on a monolayer of cultured cells covered with culture medium. To evaluate the biocompatibility of decellularized matrix glans, the following tests were performed: Metabolic Activity of Mitochondria (MTT), Lysosome Activity (Neutral Red) and quantification of genetic material (Violet Crystal). Previously the 96-well plates were seeded with a monolayer of mouse dermal fibroblasts 3T3, at a density of 1x10^3^ cells per well and incubated for 24 hours at 37°C, in a humidified 5% (v/v) CO_2_ air atmosphere with DMEM supplemented with 1% FBS. The fragments of decellularized glans matrices were incubated in fibroblast cultures at 37°C, in a humidified 5% (v/v) CO_2_, air atmosphere for 24, 48, and 72 hours. As a positive control for cytotoxic effects, dimethyl sulfoxide solution (DMSO) at 0.2% was used, and as a negative control, mouse fibroblast 3T3 cells without exposure to any external agent were used. All samples were tested in replicates of four wells (n=4), and the experiments were triplicated (n=12).

#### Seeding of mesenchymal stem cells on decellularized matrices

Fragments of the decellularized glans matrices measuring 5 x 5 x 2 mm were placed in each well of the 96-well plate. A cell suspension of mesenchymal stem cells containing 6.5x10^5^ cells/cm^2^ was seeded under static conditions in each of the fragments. After 30 minutes, 500μL of DMEM supplemented with 10% FBS was added to each well. The culture medium was changed every 2-3 days to ensure sufficient nutrients for the cells. After 7, 14, and 28 days of the static seeding, histological and biomechanical evaluations of the matrices were performed.

#### In Vitro analysis of the decellularized glans seeded with mesenchymal stem cells

The seeded matrices after 7, 14, and 28 days cultured in vitro were fixed in 10% buffered formalin, paraffin embedded, sectioned transversely (5μm), and stained with H&E. Using a light microscope interfaced with a digital image analysis system, the samples were evaluated to check the integration in vitro of mesenchymal stem cells into the decellularized glans matrices. These same samples histologically processed were now sectioned transversely (10μm), blocked for 10 minutes with a solution containing PBS, 0.2% gelatin, and 0.1% saponin, incubated for 15 minutes with DAPI (4′,6-diamidine-2-phenyl indole dihydrochloride 0.1 mg/mL, Sigma), washed twice with PBS, and mounted in buffered glycerol with 0.1 M Tris, HCl pH 8.8 and 0.01% p-fenilenodiamino. The samples were analyzed to evaluate the integration of mesenchymal stem cells to the seeded glans matrices.

#### Biomechanical testing

Fragments measuring 15x5 mm were seeded with mesenchymal stem cells at a concentration of 6.5 x 10^5^ cells/cm^2^ and maintained in culture at 37°C, in a humidified 5% (v/v) CO_2_ air atmosphere for 7, 14, and 28 days. Samples were then analyzed to evaluate the maintenance of biomechanical characteristics by seeded matrices. In biomechanical testing, specimens were tensioned longitudinally by a specific machine AME – 2KN (Oswaldo Filizola), pulling at both ends in opposite directions at a speed of 18 mm/min until breakage. The results were analyzed using software “DynaView Standard / Pro M”. The maximum force and deformation to which each sample was subjected were measured at the rupture moment. As a positive control the native tissue of the glans was used, and as a negative control, the decellularized glans matrices were used. The elasticity modulus (E = the force required to deform a sample) is defined as the ratio of tension (T) and relative deformation (Rel. Def) in this tension.
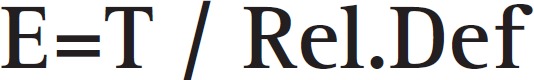



Tension (T) is the ratio between maximum force (F) and the area (SecA) of the sample. Sectional area is the product of width (W) and thickness (Tk). The relative deformation is the ratio of deformation (Df) and initial length (L0). The elasticity modulus can be rewritten as follows:
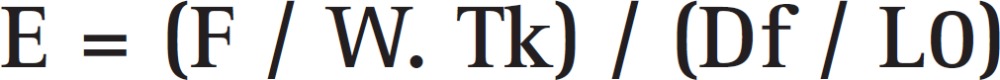



### Statistical analysis

Statistical analyses are reported as mean value ± standard deviation. To evaluate the differences between groups, two-way analyses of variance (ANOVA) for repeated measures were performed, followed by post hoc Tukey tests. For the statistical analyses of biocompatibility testing, the following nomenclature was used: group effect and interaction time versus group. Group effect means the difference between the analyzed groups, and interaction time versus group means the variation of analyzed group over time. A p-value < 0.05 was def ned as significant.

## RESULTS

### 

#### Histological evaluation

**Figure 1:**
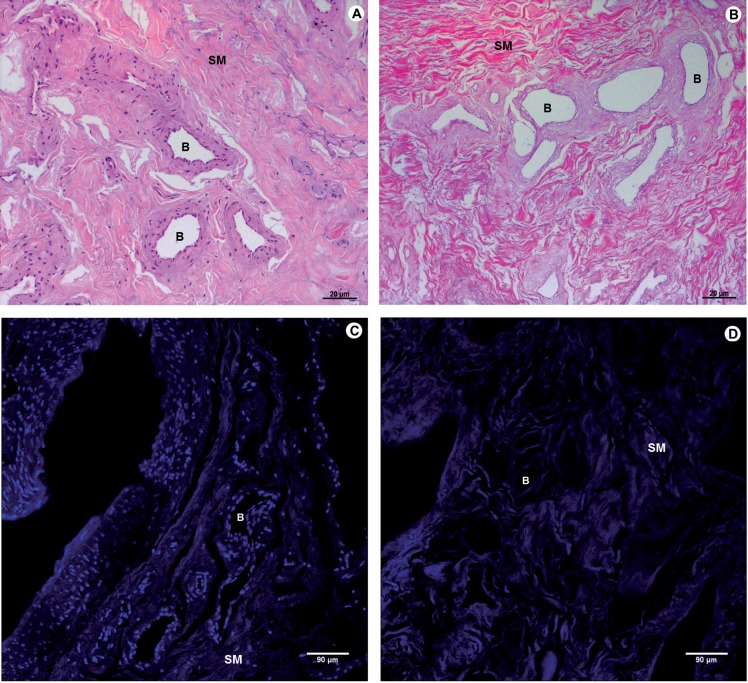
Photomicrograph of native tissue and decellularized matrix of human glans. A and C correspond to the native tissue; B and D to the decellularized matrix glans. A and B (100X) H&E and C and D (200X) DAPI. In B and D, complete decellularization of the tissue glans and the preservation of the architecture of the extracellular matrix of native tissue can be observed. SM - smooth Muscle layer B - Blood vessels.

The standardized decellularization protocol used was effective in promoting the complete removal of cellular components of the tissue after 2 weeks (Figure-1).

#### Distribution of collagen and elastic fibers

**Figure 2:**
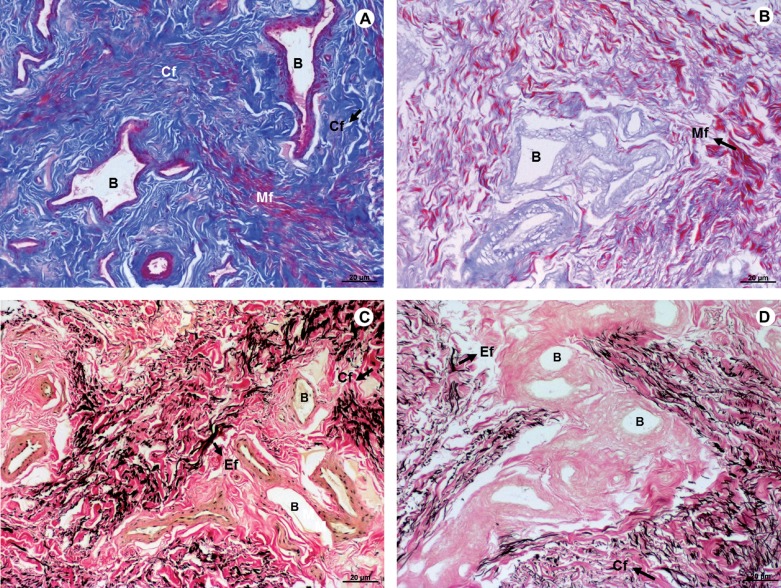
Photomicrograph of native tissue (A and C), and decellularized matrix of human glans (B and D). Masson's Trichrome – A and B; Verhoff-Van Gieson – B and D revealed the preservation of collagen fibers (blue), architecture of muscle fibers (red), and elastic fibers (black), after the decellularization process. A - D (100X). Mf – Muscle fibers; Cf – collagen fibers; Ef – Elastic fibers; B - Blood vessels.

No significant changes in the distribution of collagen were observed after the removal of the cells from native tissue. Elastic fibers were also preserved in decellularized tissue, with similar patterns to native tissue (Figure-2).

#### Cytotoxicity and biocompatibility

##### Mitochondrial metabolic activity

**Figure 3:**
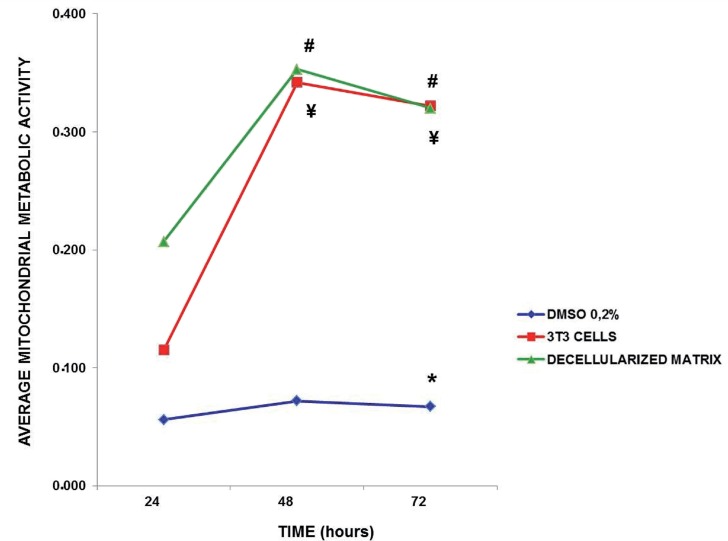
Quantification of mitochondrial metabolic activity of 3T3 cells over time “*in vitro*” culture. After 72, the 0.2% DMsO group was statistically different its 24 hour baseline time point. The 3T3 cells and decellularized glans matrices groups showed cellular respiration significantly higher at 48 and 72 hours than when analyzed with 24 hours. (* p<0.028 compared with respective 24 hour time point); (# ¥ p<0.0001 compared with respective 24 hour time point).

The metabolic activity of mitochondria of the 3T3 cells was quantified over time, which revealed a significant group effect (Figure-2; F(5.52) = 267.9; p<0.0001), showing that decellularized glans matrices group had similar patterns than 3T3 cells alone and was significantly different from DMSO group independent of the exposure time (p<0.0001 for both groups). There was an interaction time versus group effect (Figure-3; F(8.86)=9.5; p<0.0001), revealing that the decellularized glans matrices differed statistically at 48 (0.353±0.069) and 72 hours (0.320±0.032) from the values obtained at 24 hours (0.207±0.026, p<0.0001). The 3T3 cell group at 48 (0.342±0.080) and 72 hours (0.322±0.038) was statistically different from the baseline time point at 24 hours (0.115±0.018, p<0.0001). At 72 hours the 0.2% DMSO group (0.067±0.024) was statistically different from the respective baseline time at 24 hours (0.056±0.010, p<0.028) (Figure-3). The results obtained through the quantification of mitochondrial metabolic activity indicate that 3T3 cells, noncytotoxic group, and the decellularized glans matrices showed a similar pattern of cytotoxicity.

#### Neutral Red incorporation

**Figure 4:**
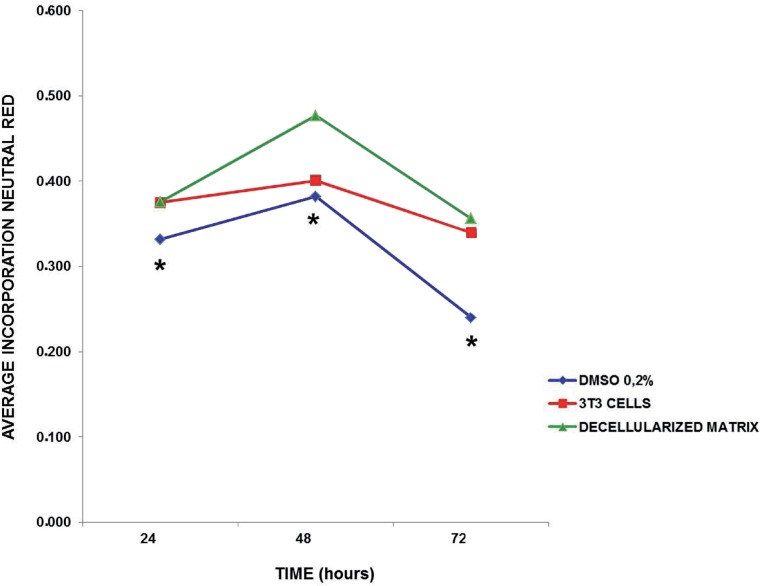
Incorporation of neutral red dye into lysosomes of 3T3 cells over time “*in vitro*” culture. The 0.2% DMsO group, independent of time, differed statistically from the 3T3 cells and decellularized glans matrices groups. (* p<0.009 for group 3T3 cells, p<0.001 for group decellularized glans matrices).

The analysis of incorporation of neutral red dye into lysosomes of viable 3T3 cells revealed a group effect (Figure-4; F(5.52)=6.8; p<0.001), showing that the decellularized glans matrices group had significant lower changes in lysosome membranes than the 0.2% DMSO group (cytotoxic group) (p<0.001) and similar to the 3T3 cells, regardless of the exposure over time (Figure-4). The results obtained showed that within 48 hours, the integrity of lysosomes membranes in 3T3 cells that were in direct contact with the decellularized glans matrices samples were better preserved when compared to the other exposure periods.

#### Quantification of genetic material – Violet Crystal dye

**Figure 5:**
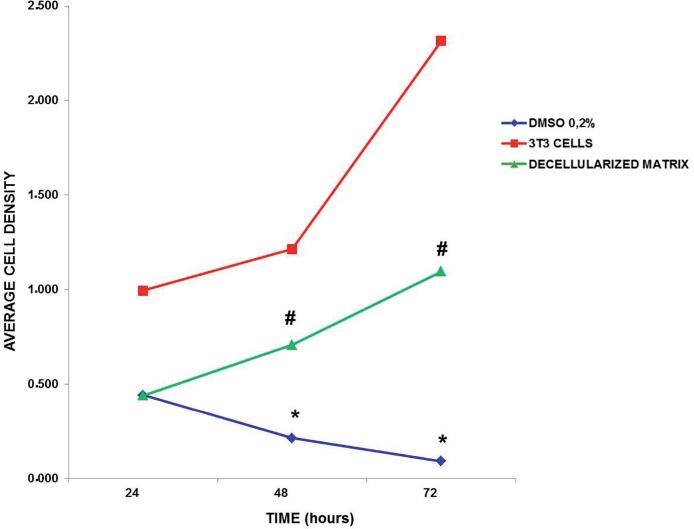
Quantification of cellular density of 3T3 cells over time *in vitro* culture. The 0.2% DMSO group has low cell density at 48 hours and the decellularized glans matrices has high cell density at 72 hours compared to their respective baseline time of 24 hours. (* p<0.026 for 48 hours; p<0.009 for 72 hours); (# p<0.009 for 48 hours and p<0.006 for 72 hours).

The analysis revealed group effect (Figure-5; F(5.52)=174.9; p<0.0001), showing that in the decellularized glans matrices and the 3T3 cells groups the cell density of viable 3T3 cells was significantly more important and different from the cell density quantified in 0.2% DMSO group (cytotoxic), (p<0.0001). The 3T3 cell group was different from the decellularized glans matrices (p<0.0001), (Figure-5).

#### Integration of mesenchymal stem cells

**Figure 6:**
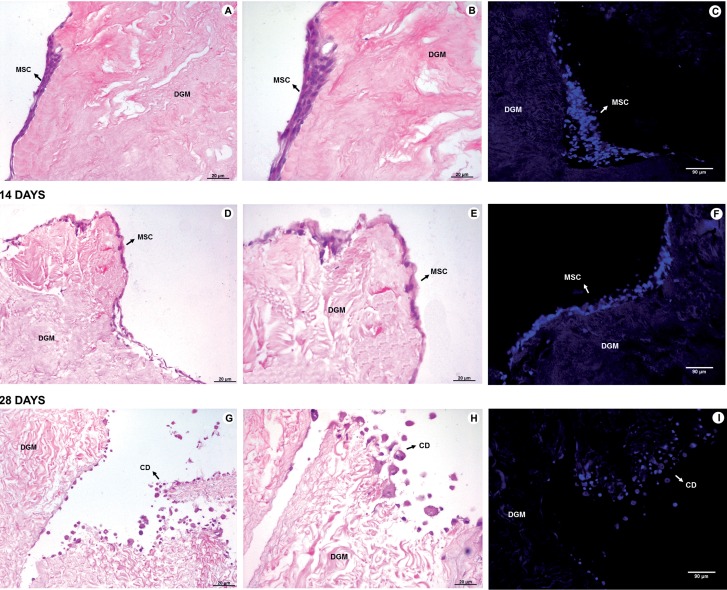
Integration of mesenchymal stem cells into decellularized glans matrices. A, B and C correspond to mesenchymal stem cells in the matrices after 7 days of in vitro culture. D, E and F 14 days of in vitro culture, and G, H and I after 28 days of in vitro culture. A, D and g (100X), and B, E and H (200X) - H&E. C, F and I (200X) - DAPI. DGM - Decellularized glans matrices; MSC - Mesenchymal stem cells, CD - cellular debris.

The static seeding of mesenchymal stem cells showed that after 7 and 14 days of in vitro culture the cells remained viable and integrated into the surface of the glans matrices (Figure-6). However, after 28 days the stem cells were no longer viable and only cellular debris were observed (Figure-6).

#### Biomechanical characteristics

**Figure 7:**
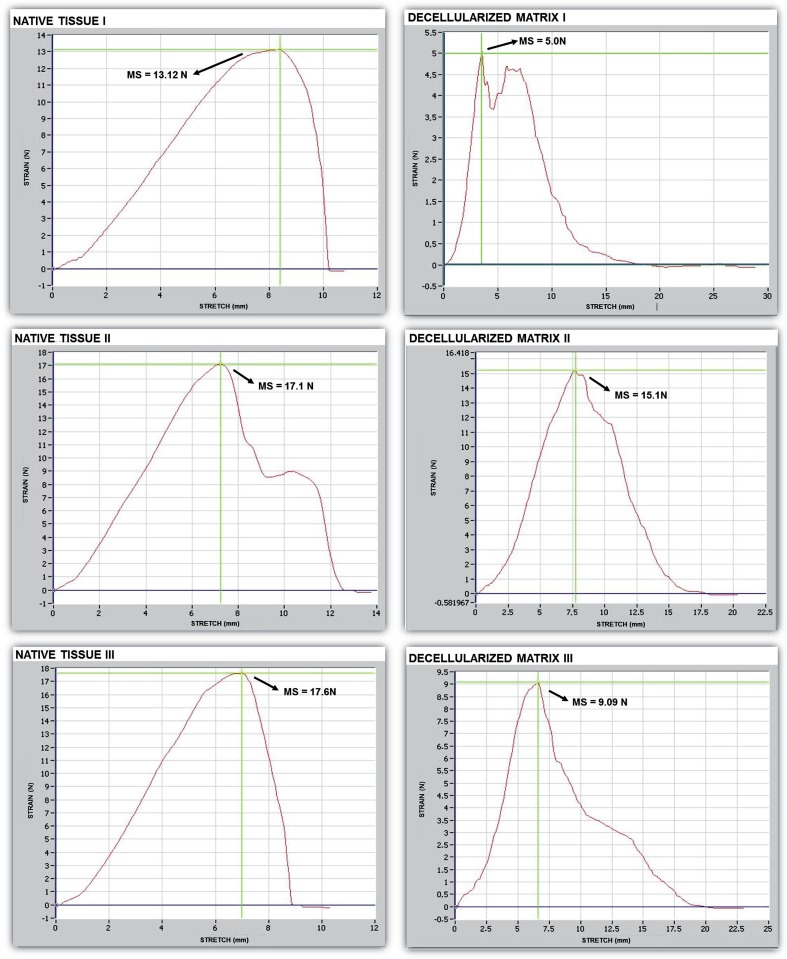
Biomechanical testing of the native tissue and decellularized glans matrices samples.

**Figure 8:**
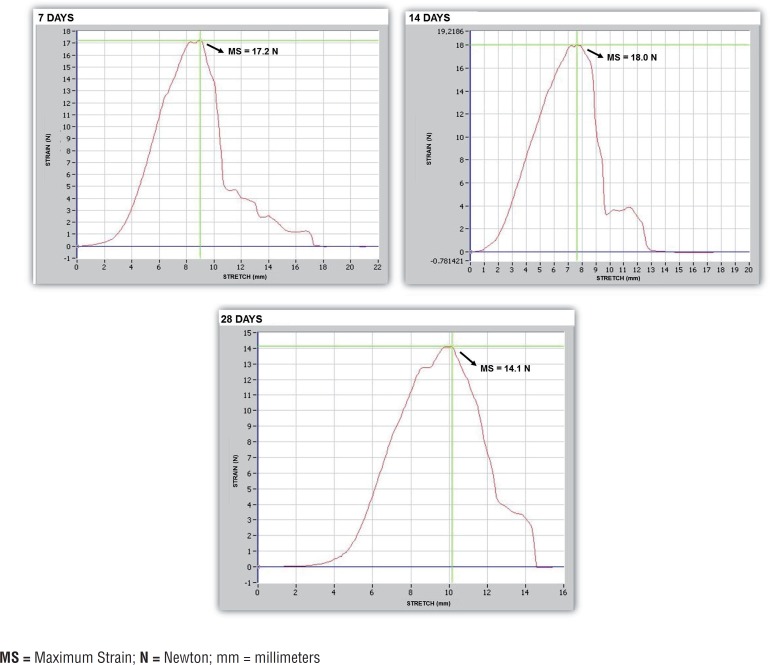
Biomechanical testing of the glans matrices after seeding with mesenchymal stem cells, and in vitro culture for 7, 14 and 28 days. **MS =** Maximum Strain; **N =** Newton; mm = millimeters

Biomechanical analysis in 7^th^ 14^th^ and 28^th^ days showed that the decellularization process caused a decrease in elastic modulus, which was 1.43Mpa in native tissue, 1.10Mpa in the seeded matrix with 14 days of culture and 0.81MPa in the decellularized matrix. A decrease in the tensile strength of the tissue samples was also observed (2.10MPa in native tissue, 1.70 Mpa in the seeded matrix with 14 days of culture and 1.03 MPa in the decellularized matrix). The specimens from decellularized matrix presented a relative deformation of 1.22, the seeded matrix with 14 days of culture presented a relative deformation of 1.50 while the specimens of native tissue also showed a relative deformation of 1.50 (Figure-7). The average maximum force and final deformation corresponded to 15.94 N and 7.5 mm for the native tissue, 18 N and 7.6mm for the seeded matrix with 14 days of culture and 9.73 N and 6.1 mm for the decellularized glans matrices (Figure-7). A reduction in maximum force required to rupture the tissue was observed at 28 days compared to 7 and 14 days of in vitro culture, which corresponds to the culture time at which cellular debris was found in the matrix (Figures 7 and 8). Overall, among the evaluated parameters, the matrix seeded with mesenchymal stem cells maintained in culture for 14 days showed an intermediate pattern of elasticity modulus and tensile strength between the values obtained with native tissue and the values with decellularized glans matrices.

## DISCUSSION

Diseases that compromise the integrity of the glans may result in aesthetic and functional changes (1). The greatest challenges when glans reconstruction is required are the limited availability of biocompatible grafts, due to its complex anatomical structure and its particular function during sexual intercourse.

Currently, the techniques for glans reconstruction are restricted to the use of autologous tissue from non-urological origin. The use of these tissues may have potential consequences such as immunological rejection, infection, or functional incompatibility between the native tissue and the implant (7). Thus, tissue engineering is a new alternative for replacing all or part of the glans (7–12). Extracellular matrices of natural origin have potential biological recognition to promote the development of new tissues (11–21).

In this study, decellularized glans matrices, after tissue processing to remove cellular components, preserved the tissue architecture and retained the biological and biomechanical properties. Analyses of the glans matrices seeded with mesenchymal stem cells exhibited the integration of these cells into the matrices, and its viability in vitro for two weeks.

We sought to establish a protocol for the decellularization of the glans matrices from cadaver that promoted minimal changes in tissue properties. The protocol described by Kwon et al. (22) was modified, and histological studies of the decellularized tissue showed effectiveness in achieving complete removal of the cellular tissue components, as well as the preservation of tissue architecture. Similarly processed natural acellular matrices from collagen have been extensively used in urogenital reconstruction (23).

Ciapetti et al. (6) showed that the potential cytocompatibility of a biomaterial must be evaluated using different quantitative methods, since the biomaterials exhibit different rates of cytotoxicity and, in addition, damages can occur in a specific structure or function of the cell. Similarly Minnem et al. (24) showed that the biocompatibility constitutes the largest problem in the development of new biodegradable materials in tissue engineering. In this experiment, we evaluated the biocompatibility of the decellularized glans matrices after 24, 48, and 72 hours of exposure to the 3T3 cells using three different methods, and found that the functionality of the mitochondria of 3T3 cells and the structural integrity of the lysosomes membranes were preserved. We also observed a high density of viable 3T3 cells. The cytotoxicity of decellularized glans matrices was therefore considered low, suggesting that they may be used for penile reconstruction.

Decellularized glans matrices and the matrices seeded with mesenchymal stem cells were also evaluated for the preservation of their biomechanical properties. The tests revealed that the elasticity modulus, tensile strength, and relative deformation of the decellularized tissue decreased compared to the native tissue. The matrices seeded with mesenchymal cells and maintained in culture for 14 days exhibited tensile modulus and tensile strength parameters values between those obtained for the native tissue and decellularized glans matrices, suggesting that once reimplanted, these matrices could be incorporated and maintain proper tension.

In this study static seeding of mesenchymal stem cells was performed on acellular glans matrices with the aim of analyzing the biological interactions between the transplanted cells and biomaterial. The matrices seeded with cells were kept in culture for 7, 14 and 28 days in order to verify the viability and integration of the stem cells to the matrices over time. It was observed that after 7 and 14 days in culture the mesenchymal stem cells remained viable and integrated into the glans matrices. After 28 days, however, only cellular debris was found, partially integrated into the matrices, suggesting that the in vitro viability of mesenchymal stem cells decreases over time. This occurs possibly due to a lack of essential supplements into culture medium, responsible for promoting the proliferation and viability of cells in culture for extended periods.

This experiment suggests that the decellularized glans matrices may be able to provide an environment conducive to the integration and viability of mesenchymal stem cells in vitro for up to 14 days, maintaining biological properties after the decellularization process. Furthermore, the mesenchymal stem cells seeded on glans matrices remained viable up to 14 days.

Further studies are needed to develop methods to obtain a higher density of mesenchymal stem cells integrated into the glans matrices and verify the tissue remodeling and reconstruction process of these matrices seeded with mesenchymal stem cells after implantation in laboratory animals.

This study haas some limitations as the reduced casuistic, the fact that is “in vitro” and the low density seeding method. Further studies are suggested in small and large animal models demonstrating how this matrix integrates “in vivo”. The vascular supply is another big challenge. Vascularization is one of the major issues in regenerative medicine. The use of decellularized tissue with preservation of the vascular pedicle and vascular growth factors like VEGF and FGF are some promising methods for overcoming this issue.

## CONCLUSIONS

The decellularization process did not alter the biological and biomechanical characteristics of the human glans matrices. When these matrices were seeded with non-autologous mesenchymal stem cells, they were able to maintain the stem cells integrity and vitality *in vitro* for up to 14 days.
